# Reference gene selection for qRT-PCR analysis of season- and tissue-specific gene expression profiles in the honey bee *Apis mellifera*

**DOI:** 10.1038/s41598-020-70965-4

**Published:** 2020-08-18

**Authors:** Ji Hyang Jeon, KyungHwan Moon, YeongHo Kim, Young Ho Kim

**Affiliations:** 1 Department of Applied Biology, Kyungpook National University, Sangju, Gyeongbuk Republic of Korea; 2 Department of Ecological Science, Kyungpook National University, Sangju, Gyeongbuk Republic of Korea

**Keywords:** Molecular biology, Transcription, Entomology

## Abstract

Honey bees are both important pollinators and model insects due to their highly developed sociality and colony management. To better understand the molecular mechanisms underlying honey bee colony management, it is important to investigate the expression of genes putatively involved in colony physiology. Although quantitative real-time PCR (qRT-PCR) can be used to quantify the relative expression of target genes, internal reference genes (which are stably expressed across different conditions) must first be identified to ensure accurate normalisation of target genes. To identify reliable reference genes in honey bee (*Apis mellifera*) colonies, therefore, we evaluated seven candidate genes (*ACT*, *EIF*, *EF1*, *RPN2*, *RPS5, RPS18* and *GAPDH*) in samples collected from three honey bee tissue types (head, thorax and abdomen) across all four seasons using three analysis programmes (NormFinder, BestKeeper and geNorm). Subsequently, we validated various normalisation methods using each of the seven reference genes and a combination of multiple genes by calculating the expression of catalase (*CAT*). Although the genes ranked as the most stable gene were slightly different on conditions and analysis methods, our results suggest that *RPS5*, *RPS18* and *GAPDH* represent optimal honey bee reference genes for target gene normalisation in qRT-PCR analysis of various honey bee tissue samples collected across seasons.

## Introduction

The Western honey bee, *Apis mellifera* L., plays an important role as a pollinator^1^. In addition, the honey bee is considered to be a key model insect due to its relatively complex behaviours, including sociality, labour division and colony management^2^. Previous studies have demonstrated that endocrine system status and gene expression are important factors for flexible honey bee colony management, which involves colonies seasonally regulating their labour division and population dynamics^3–5^. In order to extend our understanding of the molecular mechanisms that underlie the regulation of honey bee colony physiology, information on the physiological functions of the genes putatively associated with colony management can be determined by analysing their expression profiles among different seasons and honey bee tissues^6,7^.

In quantitative real-time PCR (qRT-PCR), gene-specific mRNA (or cDNA) is quantified; this method has been used extensively because of its relative speed, sensitivity, replicability and accuracy^8,9^. Therefore, qRT-PCR would be an ideal method for analysing the expression patterns of honey bee genes putatively involved in the plasticity of colony molecular physiology in samples collected across different seasons and tissues. However, because qRT-PCR results are highly sensitive to the initial amount of RNA content in the amplification reaction, the interpretation of target gene expression levels among various conditions would result in appreciable errors without the use of a reliable internal standard^7–10^. Therefore, prior to analysing target gene expression levels among conditions, reference genes are required for accurate normalisation of data to compensate for differences in the amount of RNA in various honey bee samples; these internal reference genes must show similar transcript levels across various conditions^8,9,11,12^.

Given the importance of accurate normalisation in qRT-PCR assays, reference genes have been identified and validated in various insect species^8,13,14^. According to previous studies of the honey bee, widely used reference genes were validated at different developmental stages^6^, in the brains after a bacterial challenge^15^, different ages and social roles^7,10,16^. In particular, seasonal expression stabilities of candidate reference genes have been compared between forager and nurse head in our previous study^7^. However, reference genes have yet to be compared among different honey bee tissues collected across all four seasons. In the present study, therefore, we aimed to identify the most reliable references genes among honey bee tissues types and across seasons. Specifically, we collected workers during the four seasons (i.e., spring, summer, autumn and winter) and prepared RNA samples from three tissues (head, thorax and abdomen). We then chose five candidate genes, which have been widely used as reference genes for target gene normalisation in qRT-PCR assays^6,17–19^: *β-actin* (*ACT*), *eukaryotic translation initiation factor* (*EIF*), *elongation factor 1* (*EF1*), *26S proteasome non-ATPase regulatory* (*RPN2*) and *40S ribosomal protein S5* (*RPS5*). Moreover, two genes, *40S ribosomal protein S18* (*RPS18*) and *glyceraldehyde-3-phosphate dehydrogenase* (*GAPDH*), previously identified as optimal reference genes in the honey bee head, were added in the present study^7^. Subsequently, we used three analysis programmes (NormFinder, BestKeeper and geNorm) to evaluate the expression stabilities of total seven candidate reference genes. In addition, the seven reference genes and a combination of multiple references were validated by normalising *catalase* (*CAT*) expression.

## Results

### Amplification specificity and efficiency

Prior to performing qRT-PCR, amplification specificity and efficiency were investigated. All PCR products amplified with each primer set showed a single band in 1% agarose gels and a shark single peak detected in the melting curve by RT-PCR. Furthermore, given that the forward and reverse primers for *EF1*, *RPS18* and *CAT* were designed based on two different exons containing an intron, a single band on the agarose gel and a single peak in RT-PCR observed for *EF1*,* RPS18* and *CAT* further suggested no genomic DNA contamination (Supplementary Fig. S1). In our analysis of PCR efficiencies, all seven candidate genes had linear regression coefficients (R^2^) > 0.997 and amplification efficiencies of 92–109% (Table 1).

**Table 1:** Information on the seven candidate reference genes and the target gene (*CAT*), including gene name, GenBank accession number, sequences, size, GC percentage, melting temperature of primers and amplicons.

Gene			Primers				Amplicons			
Symbol	Full gene name	Accession no	Sequence (5′ → 3′)	Size (bp)	GC (%)	TM (°C)	Size (bp)	GC (%)	Efficiency (%)	${\mathrm{R}}^{2}$
*ACT*	Beta-actin	AB023025	For. GTATGCCAACACTGTCCTTTCTG	23	48	62.9	96	46.9	103	0.999
			Rev. ATGGTGGATGGTGCTAGGGC	20	60	62.5				
*EIF*	Eukaryotic translation initiation factor 3 subunit C	XM_006564593	For. GCTGCACATGAATTTGATGCAAGAA	25	40	62.5	124	40.3	109	0.999
			Rev. CCGCGACAACATGTTCTCTCATA	23	48	62.9				
*EF1*	Elongation factor 1-alpha F2	NM_001014993	For. GTCGTGGTTATGTTGCTGGTGAT	23	48	62.9	177 (456)^a^	38.4	92	0.998
			Rev. CGCATTTCTCTTTGATATCAGCGAA	25	40	62.5				
*RPN2*	26S proteasome non-ATPase regulatory subunit 1	LOC727029	For. GTATGCGTTAGGACTTATTCATGCA	25	40	62.5	105	44.8	106	0.999
			Rev. CAACCTCCATGACGAACCATCT	22	50	62.1				
*RPS5*	40S ribosomal protein S5	XM_006570237	For. GATGTTTCTCCGTTACGACGAGT	23	48	62.9	114	44.7	92	0.999
			Rev. GAGTTCATCGGCTAAACATTCGG	23	48	62.9				
*RPS18*^b^	40S ribosomal protein S18	XM_625101	For. GATTCCCGATTGGTTTTTGAATAG	24	38	60.3	152 (446)^a^	35.5	107.6	0.999
			Rev. AACCCCAATAATGACGCAAACC	22	45	60.1				
*GAPDH*^b^	Glyceraldehyde-3-phosphate dehydrogenase	XM_393605	For. CACCTTCTGCAAAATTATGGCG	22	45	60.1	188	43.1	95.5	0.997
			Rev. ACCTTTGCCAAGTCTAACTGTTAA	24	38	60.3				
*CAT*	Apis mellifera catalase	NM_001178069	For. CTTGGCCCAAACAATCTGCAAT	22	45.5	60.3	151 (521)^a^	37.7	98	0.999
			Rev. GACATTCTCTAGGCCCACCA	20	55	60.5				

^a^Numbers in bracket indicate the size (bp) of PCR products amplified with genomic DNA.

^b^Sequence information of primers were obtained from previous study^7^.

### C_q_ distributions of reference genes

**Figure 1:**
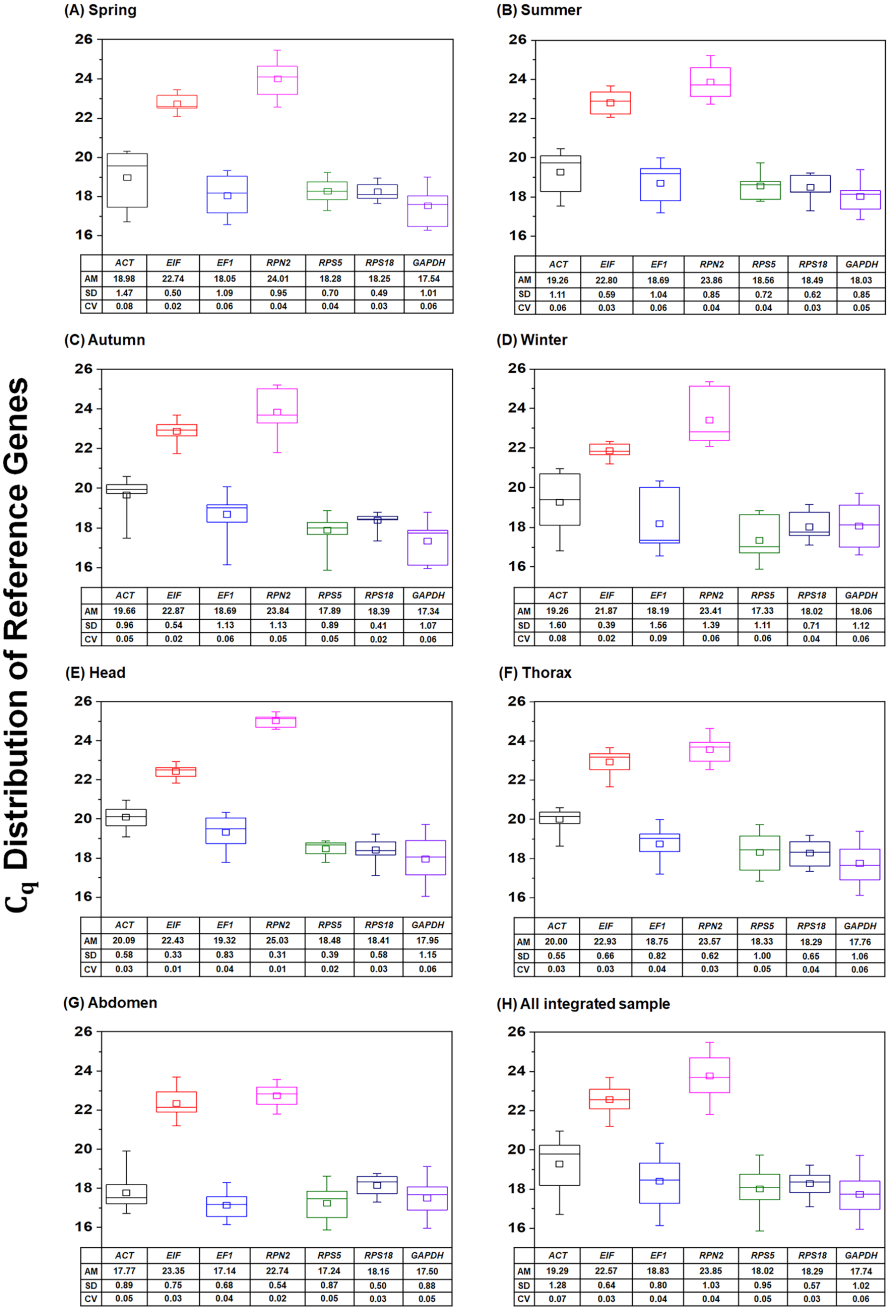
Box plot comparisons of C_q_ values for the seven candidate reference genes in honey bee samples. Samples were prepared from four seasons (**A**–**D**), three tissue types (**E**–**G**) and an integration of all samples (**H**). The horizontal lines in the box indicate the 25th, 50th and 75th percentile values. The square symbol in the big box shows the mean median. The error bars denote the maximum and minimum values.

Expression levels, as indicated by C_q_ values, of the seven candidate reference genes in honey bee transcript samples prepared from four seasons and three tissue types were analysed (Fig. 1). Based on arithmetic means (AM) and standard deviation (SD) values, coefficient of variation (CV) values were calculated as follows: CV = SD/AM. Across the four seasons, *EIF* showed the lowest variability with a coefficient of variation (CV) of 0.02–0.03 (Fig. 1A–D). Among the three tissue types, the CV values of *RPN2* were the lowest of the seven genes (0.01 in the head, 0.03 in the thorax and 0.02 in the abdomen) (Fig. 1E–G). *EIF* was also the most stable gene in the head and thorax (Fig. 1E,F). As shown in Fig. 1H, by comparison of the seven genes’ C_q_ values obtained from all samples among the various seasons and tissue types, *EIF* and *RPS18* were the least variable gene (CV = 0.03).

### Analysis of expression stability using three program

#### NormFinder analysis

**Figure 2:**
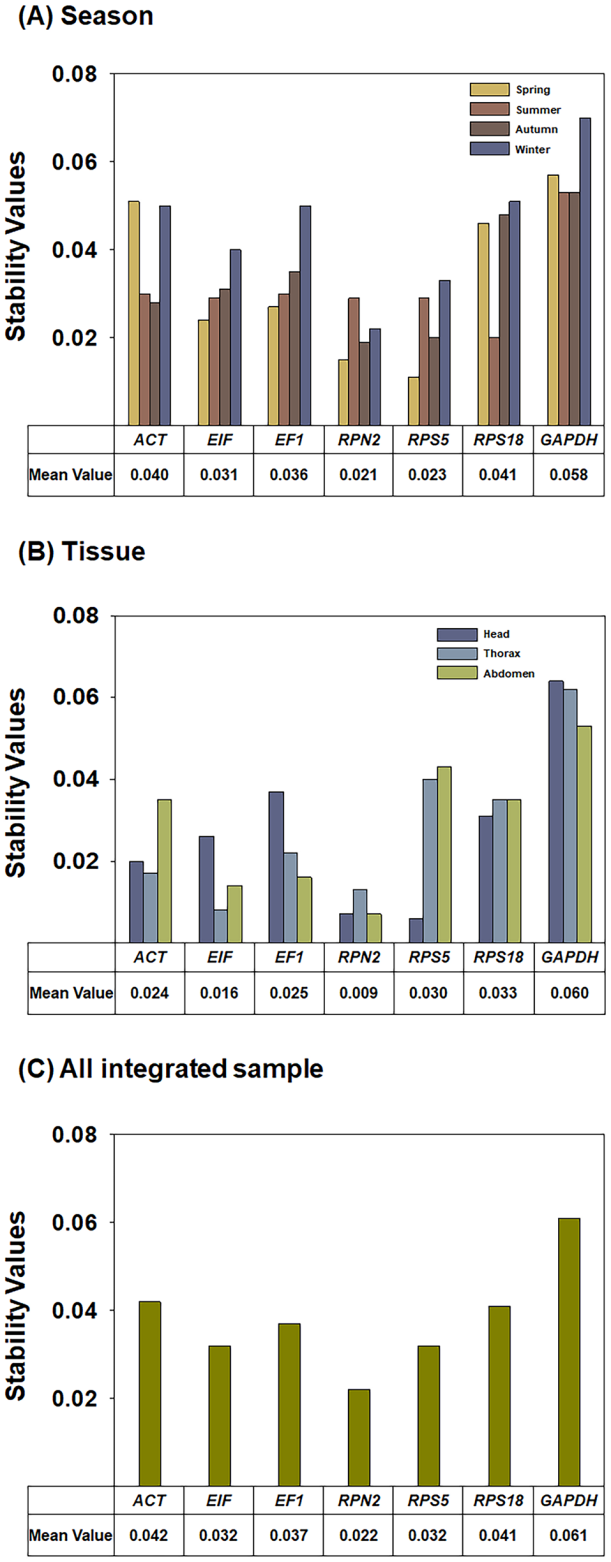
Expression stability values of the seven candidate honey bee reference genes calculated by NormFinder. Average stability values (mean values) were arithmetically calculated from honey bee samples prepared from four seasons (**A**), three tissue types (**B**) and an integration of all samples (**C**).

Based on the expression variation of candidate genes, NormFinder identifies the optimal reference gene by calculating stability values^20,21^. According to average stability values (mean values), which were arithmetically calculated for the four seasons, *RPN2* was the most stable gene (mean value = 0.021) (Fig. 2A). In a comparison of all seven genes’ stability values, *RPS5* was the most stable gene in spring. *RPS18* was the most stable gene in summer, whereas *RPN2* was the most stable gene in autumn and winter (Fig. 2A, Table 2). In gene stability analysis of the three tissue types, *RPN2* was the most stable (mean stability = 0.009) (Fig. 2B). In the stability analysis of specific tissue types, *RPS5* was highest ranked gene in the head (least stable), while the most stable expression levels in thorax and abdomen were *EIF* and *RPN2*, respectively (Fig. 2B, Table 2). When the stability values of genes were calculated by combining all four seasons and three tissue types, the stability rank from the most (lowest value) to least (highest value) stable was as follows: *RPN2* > *EIF* = *RPS5* > *EF1* > *RPS18* > *ACT* > *GAPDH* (Fig. 2C, Table 2).

**Table 2:** Summary of gene expression stability values analysed by C_q_ distribution in NormFinder, BestKeeper and geNorm.

Rank	Spring				Summer				Autumn				Winter			
	*C*_*q*_ (CV)^a^	NormFinder (SV)^b^	BestKeeper (SD)^c^	geNorm (M value)	*C*_*q*_ (CV)	NormFinder (SV)	BestKeeper (SD)	geNorm (M value)	*C*_*q*_ (CV)	NormFinder (SV)	BestKeeper (SD)	geNorm (M value)	*C*_*q*_ (CV)	NormFinder (SV)	BestKeeper (SD)	geNorm (M value)
1	*EIF* (0.02)*	*RPS5* (0.011)*	*EIF* (0.41)*	*EIF* (0.207)**	*RPS18* (0.03)*	*RPS18* (0.020)*	*RPS18* (0.48)*	*EF1* (0.32)**	*RPS18* (0.02)*	*RPN2* (0.019)*	*RPS18* (0.24)*	*RPS5* (0.324)**	*EIF* (0.02)*	*RPN2* (0.022)*	*EIF* (0.31)*	*RPN2* (0.265)**
2	*RPS18* (0.03)*	*RPN2* (0.015)*	*RPS18* (0.43)*	*RPS5* (0.23)**	*EIF* (0.03)*	*RPS5* (0.029)*	*EIF* (0.51)*	*ACT* (0.346)**	*EIF* (0.02)*	*RPS5* (0.020)*	*EIF* (0.38)*	*EF1* (0.347)**	*RPS18* (0.04)*	*RPS5* (0.033)*	*RPS18* (0.59)*	*EF1* (0.302)**
3	*RPS5* (0.04)*	*EIF* (0.024)*	*RPS5* (0.58)*	*RPS18* (0.238)**	*RPS5* (0.04)*	*RPN2* (0.029)*	*RPS5* (0.58)*	*RPS18* (0.422)**	*RPS5* (0.05)*	*ACT* (0.028)*	*RPS5* (0.61)*	*ACT* (0.377)**	*RPN2* (0.06)*	*EIF* (0.040)*	*GAPDH* (0.92)*	*RPS5* (0.347)**
4	*RPN2* (0.04)*	*EF1* (0.027)*	*RPN2* (0.77)*	*EF1* (0.429)**	*RPN2* (0.04)*	*EIF* (0.029)*	*GAPDH* (0.68)*	*RPS5* (0.567)*	*ACT* (0.05)*	*EIF* (0.031)*	*ACT* (0.69)*	*RPN2* (0.512)*	*RPS5* (0.06)*	*EF1* (0.050)*	*RPS5* (0.93)*	*RPS18* (0.498)**
5	*EF1* (0.06)*	*RPS18* (0.046)*	*GAPDH* (0.79)*	*RPN2* (0.566)*	*GAPDH* (0.05)*	*EF1* (0.030)*	*RPN2* (0.69)*	*EIF* (0.65)*	*RPN2* (0.05)*	*EF1* (0.035)*	*EF1* (0.80)*	*RPS18* (0.632)*	*GAPDH* (0.06)*	*ACT* (0.050)*	*RPN2* (1.21)	*ACT* (0.63)*
6	*GAPDH* (0.06)*	*ACT* (0.051)*	*EF1* (0.92)*	*ACT* (0.704)*	*EF1* (0.06)*	*ACT* (0.030)*	*EF1* (0.91)*	*RPN2* (0.74)*	*EF1* (0.06)*	*RPS18* (0.048)*	*RPN2* (0.86)*	*EIF* (0.709)*	*ACT* (0.08)*	*RPS18* (0.051)*	*EF1* (1.32)	*GAPDH* (0.773)*
7	*ACT* (0.08)*	*GAPDH* (0.057)*	*ACT* (1.27)	*GAPDH* (0.987)*	*ACT* (0.06)*	*GAPDH* (0.053)*	*ACT* (0.95)*	*GAPDH* (0.925)*	*GAPDH* (0.06)*	*GAPDH* (0.053)*	*GAPDH* (0.88)*	*GAPDH* (0.917)*	*EF1* (0.09)*	*GAPDH* (0.070)*	*ACT* (1.36)	*EIF* (0.914)*

Rank	Head				Thorax				Abdomen				All integrated sample			
	*C*_*q*_ (CV)	NormFinder (SV)	BestKeeper (SD)	geNorm (M value)	*C*_*q*_ (CV)	NormFinder (SV)	BestKeeper (SD)	geNorm (M value)	*C*_*q*_ (CV)	NormFinder (SV)	BestKeeper (SD)	geNorm (M value)	*C*_*q*_ (CV)	NormFinder (SV)	BestKeeper (SD)	geNorm (M value)
1	*RPN2* (0.01)*	*RPS5* (0.006)*	*RPN2* (0.26)*	*RPS5* (0.118)**	*EIF* (0.03)*	*EIF* (0.008)*	*ACT* (0.40)*	*EIF* (0.172)**	*RPN2* (0.02)*	*RPN2* (0.007)*	*RPN2* (0.44)*	*RPS18* (0.234)**	*RPS18* (0.03)*	*RPN2* (0.022)*	*RPS18* (0.47)*	*ACT* (0.252)**
2	*EIF* (0.01)*	*RPN2* (0.007)*	*EIF* (0.27)*	*RPN2* (0.123)**	*RPN2* (0.03)*	*RPN2* (0.013)*	*RPN2* (0.50)*	*RPS18* (0.19)**	*RPS18* (0.03)*	*EIF* (0.014)*	*RPS18* (0.44)*	*RPN2* (0.236)**	*EIF* (0.03)*	*EIF* (0.032)*	*EIF* (0.53)*	*EF1* (0.254)**
3	*RPS5* (0.02)*	*ACT* (0.020)*	*RPS5* (0.34)*	*RPS18* (0.141)**	*ACT* (0.03)*	*ACT* (0.017)*	*EIF* (0.53)*	*EF1* (0.205)**	*EIF* (0.03)*	*EF1* (0.016)*	*EF1* (0.54)*	*EIF* (0.256)**	*RPN2* (0.04)*	*RPS5* (0.032)*	*RPS5* (0.76)*	*RPS18* (0.276)**
4	*RPS18* (0.03)*	*EIF* (0.026)*	*RPS18* (0.42)*	*ACT* (0.241)**	*RPS18* (0.04)*	*EF1* (0.022)*	*RPS18* (0.56)*	*RPN2* (0.27)**	*EF1* (0.04)*	*RPS18* (0.035)*	*EIF* (0.61)*	*EF1* (0.281)**	*EF1* (0.04)*	*EF1* (0.037)*	*GAPDH* (0.82)*	*RPN2* (0.348)**
5	*ACT* (0.03)*	*RPS18* (0.031)*	*ACT* (0.45)*	*GAPDH* (0.35)**	*EF1* (0.04)*	*RPS18* (0.035)*	*EF1* (0.63)*	*ACT* (0.328)**	*ACT* (0.05)*	*ACT* (0.035)*	*ACT* (0.66)*	*GAPDH* (0.344)**	*RPS5* (0.05)*	*RPS18* (0.041)*	*RPN2* (0.92)*	*EIF* (0.399)**
6	*EF1* (0.04)*	*EF1* (0.037)*	*EF1* (0.67)*	*EF1* (0.425)**	*RPS5* (0.05)*	*RPS5* (0.040)*	*RPS5* (0.83)*	*RPS5* (0.398)**	*GAPDH* (0.05)*	*RPS5* (0.043)*	*GAPDH* (0.69)*	*ACT* (0.415)**	*GAPDH* (0.06)*	*ACT* (0.042)*	*EF1* (1.03)	*RPS5* (0.421)**
7	*GAPDH* (0.06)*	*GAPDH* (0.064)*	*GAPDH* (0.96)*	*EIF* (0.494)**	*GAPDH* (0.06)*	*GAPDH* (0.062)*	*GAPDH* (0.83)*	*GAPDH* (0.526)*	*RPS5* (0.05)*	*GAPDH* (0.053)*	*RPS5* (0.70)*	*RPS5* (0.513)*	*ACT* (0.07)*	*GAPDH* (0.061)*	*ACT* (1.09)	*GAPDH* (0.46)**

^a^CV refers to the coefficient of variation analysed by C_q_ distribution as indicated in Fig. 1

^b^SV refers to the stability values analysed by NormFinder.

^c^SD indicates the standard deviation of C_q_ values analysed by BestKeeper.

*Stability values of the genes below the criteria CV < 1 (C_q_ distribution analysis), SV < 0.15 (NormFinder), SD < 1.0 (BestKeeper) and M < 1.5 (geNorm).

**Stability values of the genes below the criterion M < 0.5 (geNorm).

#### BestKeeper analysis

Genes that show low SD (usually < 1) and CV values can be chosen as the more stable reference genes in the BestKeeper algorithm^22,23^. Based on SD and CV values, BestKeeper highlighted *EIF* (in spring and winter) and *RPS18* (in summer and autumn) as the most appropriate reference gene with the least C_q_ variation (Table 3). Across the three tissue types, according to SD and CV scores, *RPN2* was the top ranked gene in the head and abdomen (least stable), while *ACT* was identified as the optimal reference gene in the thorax (Table 3). Although the stability of genes in the head, thorax and abdomen were variable, all seven genes had SD < 1.0 in all tissues, which suggests that any of the genes could be used as a reference gene for normalisation of target gene expression in the head, thorax or abdomen of honey bees (Table 3). When the C_q_ values of the seven genes were combined across seasons and tissue types, BestKeeper revealed that *RPS18*, *EIF*, *RPS5, GAPDH* and *RPN2* had SD values < 1.0; thus, these genes are perhaps the best candidates as reference genes (Table 3, see All integrated sample).

**Table 3:** Gene expression stability values of the seven candidate reference genes analysed by BestKeeper.

Rank	Spring						Summer						Autumn						Winter					
	Gene	SD^a^	CV^b^	GM (C_q_)^c^	CD (R^2^)^d^	P value	Gene	SD	CV	GM (C_q_ )	CD (R^2^)	P value	Gene	SD	CV	GM (C_q_ )	CD (R^2^)	P value	Gene	SD	CV	GM (C_q_ )	CD (R^2^)	P value
1	*EIF*	0.41	1.82	22.74	0.75	0.02	*RPS18*	0.48	2.62	18.48	0.80	0.01	*RPS18*	0.24	1.33	18.38	− 0.03	0.95	*EIF*	0.31	1.41	21.86	0.53	0.14
2	*RPS18*	0.43	2.34	18.24	0.19	0.63	*EIF*	0.51	2.22	22.79	0.55	0.12	*EIF*	0.38	1.67	22.87	0.55	0.12	*RPS18*	0.59	3.27	18.01	0.39	0.30
3	*RPS5*	0.58	3.18	18.27	0.90	0.00	*RPS5*	0.58	3.12	18.55	0.72	0.03	*RPS5*	0.61	3.39	17.87	0.93	0.00	*GAPDH*	0.92	5.12	18.03	0.37	0.33
4	*RPN2*	0.77	3.19	24.00	0.88	0.00	*GAPDH*	0.68	3.75	18.01	0.36	0.35	*ACT*	0.69	3.50	19.63	0.84	0.00	*RPS5*	0.93	5.39	17.30	0.88	0.00
5	*GAPDH*	0.79	4.48	17.51	0.52	0.15	*RPN2*	0.69	2.89	23.84	0.65	0.06	*EF1*	0.80	4.26	18.66	0.92	0.00	*RPN2*	1.21	5.17	23.37	0.91	0.00
6	*EF1*	0.92	5.10	18.02	0.97	0.00	*EF1*	0.91	4.86	18.67	0.95	0.00	*RPN2*	0.86	3.61	23.81	0.91	0.00	*EF1*	1.32	7.28	18.13	0.93	0.00
7	*ACT*	1.27	6.67	18.93	0.97	0.00	*ACT*	0.95	4.94	19.23	0.98	0.00	*GAPDH*	0.88	5.09	17.31	0.68	0.05	*ACT*	1.36	7.08	19.20	0.93	0.00

Rank	Head						Thorax						Abdomen						All integrated sample					
	Gene	SD^a^	CV^b^	GM (C_q_)^c^	CD (R^2^)^d^	P value	Gene	SD	CV	GM (C_q_ )	CD (R^2^)	P value	Gene	SD	CV	GM (C_q_ )	CD (R^2^)	P value	Gene	SD	CV	GM (C_q_ )	CD (R^2^)	P value
1	*RPN2*	0.26	1.03	25.02	0.74	0.01	*ACT*	0.40	1.99	20.00	0.77	0.00	*RPN2*	0.44	1.95	22.74	0.86	0.00	*RPS18*	0.47	2.57	18.28	0.42	0.01
2	*EIF*	0.27	1.21	22.43	− 0.16	0.63	*RPN2*	0.50	2.11	23.56	0.81	0.00	*RPS18*	0.44	2.42	18.15	0.33	0.29	*EIF*	0.53	2.35	22.56	0.57	0.00
3	*RPS5*	0.34	1.85	18.47	0.78	0.00	*EIF*	0.53	2.30	22.92	0.91	0.00	*EF1*	0.54	3.13	17.13	0.91	0.00	*RPS5*	0.76	4.22	17.99	0.83	0.00
4	*RPS18*	0.42	2.27	18.40	0.45	0.14	*RPS18*	0.56	3.06	18.28	0.53	0.08	*EIF*	0.61	2.71	22.34	0.87	0.00	*GAPDH*	0.82	4.64	17.71	0.43	0.01
5	*ACT*	0.45	2.25	20.08	0.68	0.02	*EF1*	0.63	3.35	18.74	0.91	0.00	*ACT*	0.66	3.73	17.76	0.81	0.00	*RPN2*	0.92	3.85	23.75	0.84	0.00
6	*EF1*	0.67	3.44	19.31	0.68	0.02	*RPS5*	0.83	4.55	18.30	0.81	0.00	*GAPDH*	0.69	3.93	17.48	0.39	0.21	*EF1*	1.03	5.62	18.37	0.93	0.00
7	*GAPDH*	0.96	5.33	17.92	0.61	0.03	*GAPDH*	0.83	4.70	17.73	0.42	0.17	*RPS5*	0.70	4.05	17.22	0.67	0.02	*ACT*	1.09	5.65	19.25	0.89	0.00

^a^SD indicates the standard deviation of C_q_ values.

^b^CV refers to the coefficient of variation value.

^c^GM represents the geometric mean of C_q_ values.

^d^CD indicates the coefficient of determination value.

#### geNorm analysis

**Figure 3:**
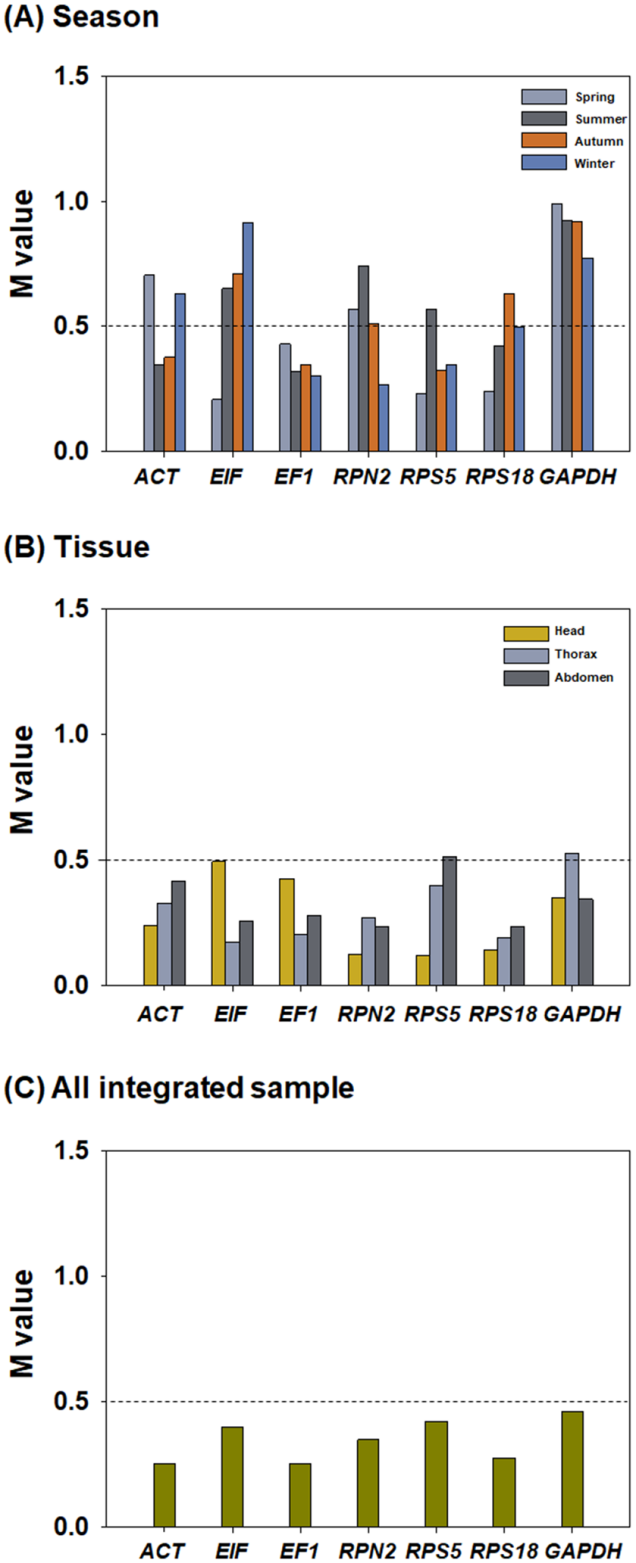
Expression stability values (M) of the seven candidate honey bee reference genes calculated by geNorm. Samples were prepared from four seasons (**A**), three tissue types (**B**) and an integration of all samples (**C**). The dotted lines indicate the M = 0.5 value, which is the criterion for appropriate reference gene selection.

The average expression stability values (M values) of the seven candidates were also determined using geNorm across the different seasons and tissue types (Fig. 3). M ≤ 0.5 has been suggested as the criterion for appropriate reference gene selection^21,24^. In the seasonal comparison, the M values of *EF1* were < 0.5 in each of the seasons, whereas the other six genes had M ≥ 0.5 in at least one season (Fig. 3A); consequently, *EF1* was perhaps the most suitable reference gene for target gene normalisation when analysing seasonal gene expression trends in honey bees. When the M scores of candidate reference genes were compared across tissue types, all genes had M ≤ 0.5 with the exception of *GAPDH* and *RPS5* in the thorax and abdomen, respectively (Fig. 3B); hence, *ACT*, *EIF*, *EF1*, *RPN2* and *RPS18* may be useful as reference genes for gene expression analysis in different honey bee tissue types. When the C_q_ values of the seven genes obtained from different seasons and tissue types were combined, the M values of all genes were < 0.5 (Fig. 3C), suggesting that any one of the seven genes could be a reference gene according to geNorm analysis.

**Figure 4:**
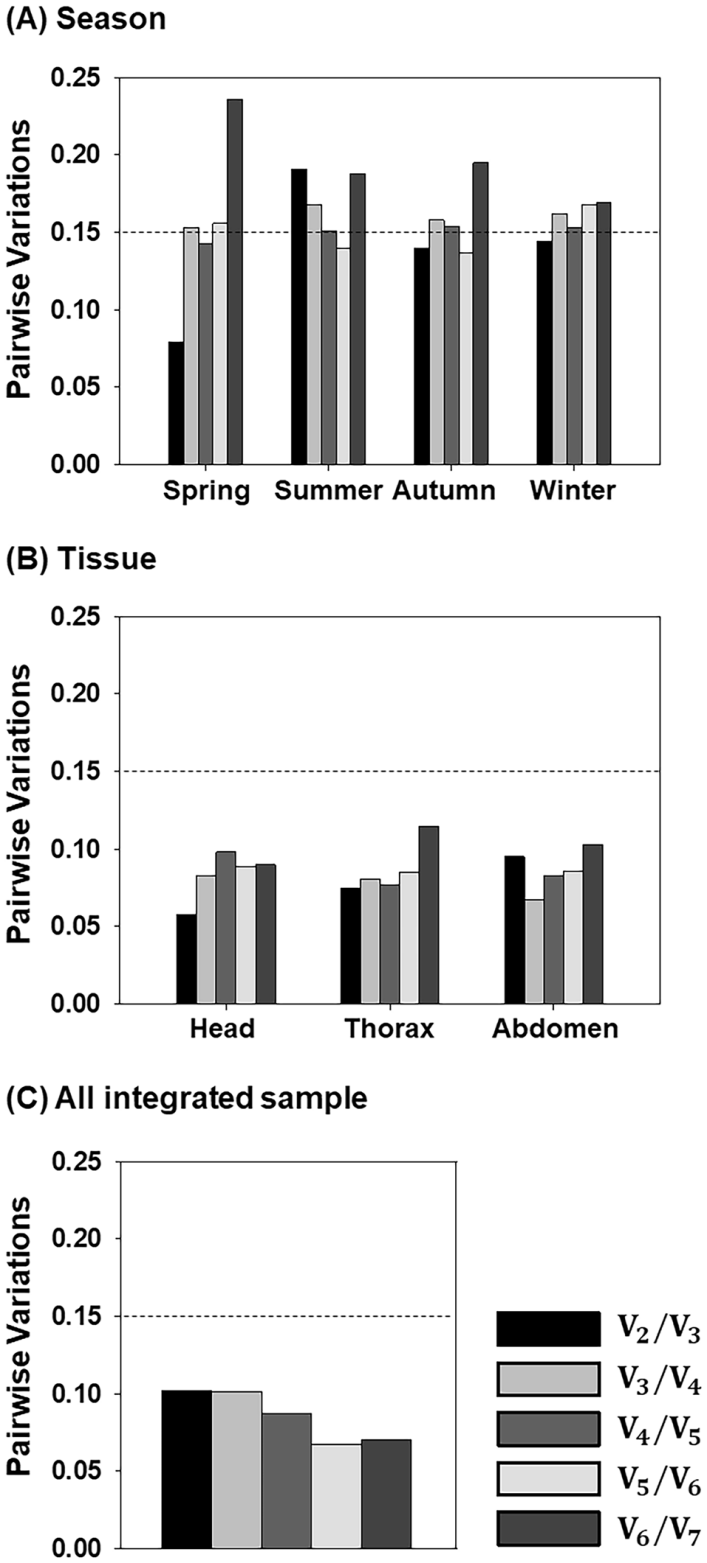
geNorm pairwise variation analysis was used to determine the optimal number of references for target gene normalisation. Pairwise variation values (V_n_/V_n+1_) were calculated from honey bee samples prepared from four seasons (**A**), three tissue types (**B**) and an integration of all samples (**C**). The dotted lines indicate where the pairwise variation = 0.15, which was the cutoff value used to indicate the optimal number of reference genes.

In additional analysis, geNorm was used to calculate pairwise variation (V_n_/V_n+1_) values that would indicate the optimal number of reference genes for target gene normalisation. According to previous studies, 0.15 is a suitable cutoff value in pairwise variation analysis^25^. In seasonal analysis, the V_2_/V_3_ values of spring, autumn and winter bees were 0.079, 0.14 and 0.144, respectively, indicating that a combination of two genes (*EIF* + *RPS5* for spring; *RPS5* + *EF1* for autumn; *RPN2* + *EF1* for winter) would be sufficient for target gene normalisation in these seasons (Fig. 4A), based on the ranks of gene expression stability analysed by geNorm (shown in Fig. 3A, Table 2). In contrast, only V_5_/V_6_ of summer was under the 0.15 cutoff value (V_5_/V_6_ = 0.14) (Fig. 4A), implying that at least five reference genes should be combined for normalising target gene expression in summer bee. Similarly, pairwise variation analysis demonstrated that V_2_/V_3_ values were lower than the cutoff value in head, thorax and abdomen, which also suggests that two genes (*RPS5* + *RPN2* for the head; *EIF* + *RPS18* for the thorax; and *RPS18* + *RPN2* for the abdomen) would be sufficient for calculating gene expression (Fig. 4B). When all the samples were combined, pairwise variation analysis revealed that V_2_/V_3_ values were higher than V_3_/V_4_, V_4_/V_5_, V_5_/V_6_ and V_6_/V_7_ values; however, V_2_/V_3_ was 0.102, which was still under the 0.15 cutoff value. This finding supports the conclusion that two genes, *ACT* and *EF1*, were the optimal normalisation factors for gene expression analysis (Fig. 4C).

### Reference gene validation

**Figure 5:**
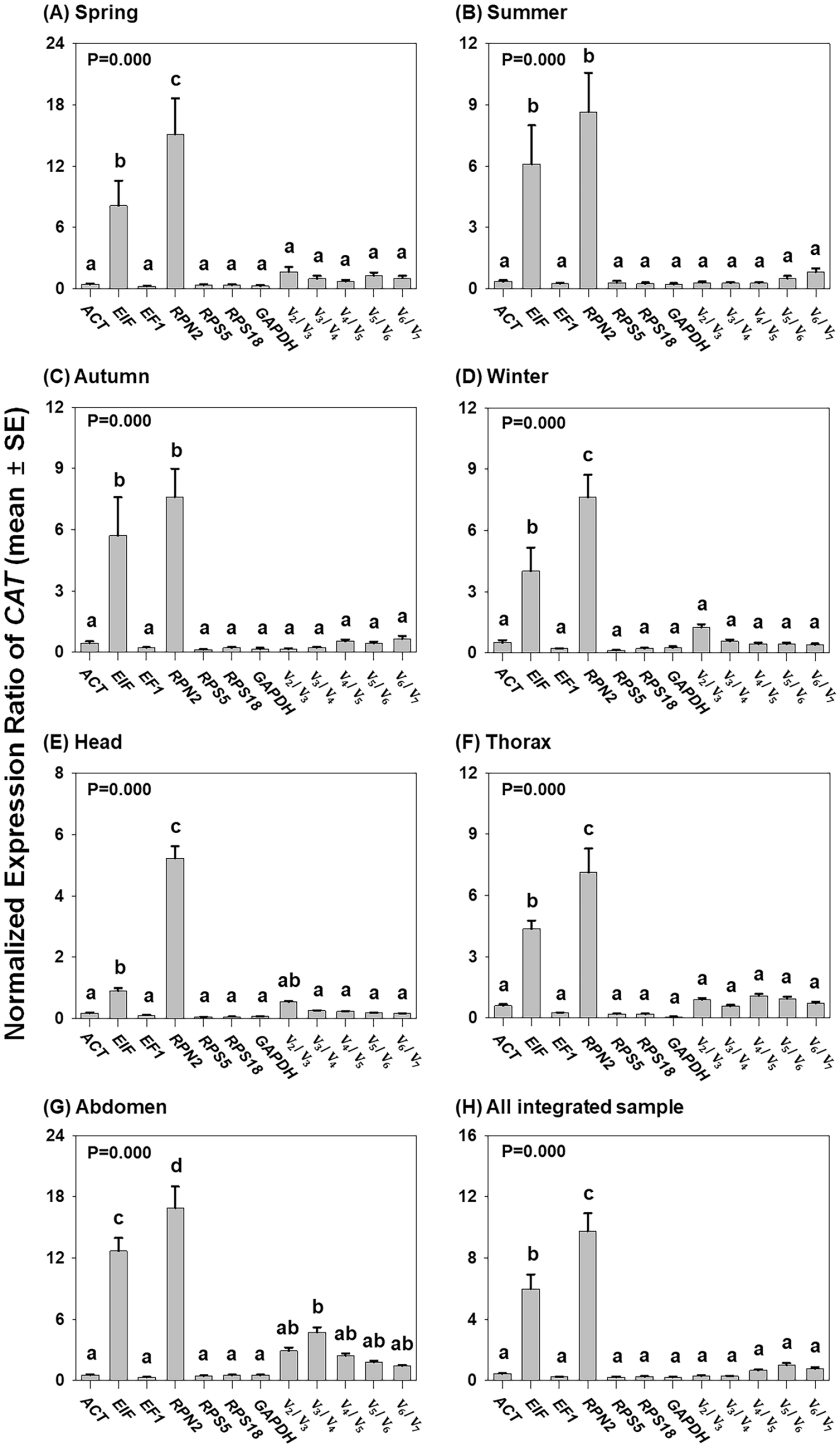
Comparison of expression levels of *CAT* in honey bee samples normalised with a single gene from the seven references and a combination of multiple reference genes. Samples were prepared from four seasons (**A**–**D**), three tissue types (**E**–**G**) and the integration of all samples (**H**). The expression levels of *CAT* normalised with different methods were statistically analysed with a one-way ANOVA followed by Tukey’s multiple comparison post-hoc test and different letters indicate significantly different values (P < 0.05). Data are presented as mean values ± SE.

Since geNorm pairwise analysis suggested application of multiple reference genes for target gene normalisation (Fig. 4), we compared expression levels of *CAT* (as the target gene) normalised with each of the seven reference genes and multiple reference genes across the four seasons and three tissue types (Fig. 5). In seasonal analysis, the number of selected reference genes did not alter the expression levels of *CAT* (Fig. 5A–D). Furthermore, *CAT* expression levels normalised with a single gene (i.e., *ACT*, *EF1*, *RPS5*, *RPS18* or *GAPDH*) were not significantly different from *CAT* expression levels normalised with multiple reference genes (P = 1.000 for spring, summer and autumn; P = 0.868 for winter) (Fig. 5A–D). These results indicate that a single gene, namely *ACT*, *EF1*, *RPS5, RPS18* or *GAPDH,* could be used as a reference when analysing seasonal expression trends of target genes in honey bees. In a comparison of *CAT* expression in the three tissue types, when *ACT*, *EF1*, *RPS5*, *RPS18* or *GAPDH* were used as single reference genes, *CAT* expression levels were not significantly different from those obtained with a combination of multiple reference genes in the head (P = 0.169), thorax (P = 0.720) and abdomen (P = 0.379) analysis (Fig. 5E–G). We also compared the overall expression levels of *CAT* normalised with a single candidate reference gene and a multiple gene combination (Fig. 5H). Analysis showed that the expression levels of *CAT* normalised with any number of multiple gene combinations were not significantly different. In addition, each of the *ACT*, *EF1*, *RPS5*, *RPS18* and *GAPDH* normalisations had expression levels of *CAT* that were statistically similar to those obtained with multiple gene combinations (P = 0.981) (Fig. 5H). This suggests that it would be possible to use a single gene, *ACT*, *EF1*, *RPS5*, *RPS18* or *GAPDH*, as the optimal reference gene for target gene normalisation across different seasons and honey bee tissue types.

## Discussion

In order to find optimal reference genes for qRT-PCR assay in the honey bee across four seasons and three tissue types, we evaluated the expression stabilities of seven candidate reference genes, *ACT*, *EIF*, *EF1*, *RPN2*, *RPS5*, *RPS18* and *GAPDH*, using three analysis programmes: NormFinder, BestKeeper and geNorm. In addition, pairwise variation analysis in geNorm was used to identify the optimal number of honey bee reference genes required for normalisation of target gene expression. The normalisation methods, including individual reference genes or combinations of multiple genes, were also validated by analysing *CAT* expression in the various samples.

Consistent with observations in previous studies^7,12,13^, the three algorithms, NormFinder, BestKeeper and geNorm, produced different results when ranking gene stability in the present study; therefore, the combined use of all algorithms would ensure more credible results^13^. Considering the cutoff values in each algorithm, most of the seven candidate genes could be deemed acceptable for use as reference genes when analysing gene expression in different conditions in honey bees. Although most previous studies did not set a cutoff value for gene stability in NormFinder analysis^6–8,14–16,21,22^, several recent studies have suggested values < 0.15 as a suitable cutoff^20,26,27^. Based on this criterion, all seven genes were suitable reference genes according to NormFinder. This result is supported by the distribution of C_q_ values, which indicated that all seven candidate genes were stably expressed with CV values < 1, which is considered to indicate low variance^28^. In BestKeeper analysis, all seven genes were also determined to be appropriate reference genes for analysis of honey bee gene expression in summer, autumn and among the three tissue types as indicated by SD values < 1^22,23^. In contrast, some genes (*ACT* in spring; *RPN2*, *EF1* and *ACT* in winter; *EF1* and *ACT* in integrated sample) can not be suggested to be an optimal reference gene due to their SD values > 1. When considering M ≤ 0.5 as the criterion for suitable reference gene selection in geNorm analysis^21,24^, all seven genes were acceptable references only in the head and the integration of all samples. In contrast, when using M ≤ 1.5 as the criterion, which has been widely suggested as an acceptable level for reference gene selection^22,25^, all seven genes could be regarded as reference genes across seasons and tissue types. Taken together, our combined analyses suggest that *EIF*, *RPS5*, *RPS18* and *GAPDH* would be most suitable as optimal reference genes for the normalisation of target gene expression in honey bee samples prepared from a variety of tissues across seasons.

In addition to the measurement of gene expression stabilities, geNorm can be used to adjunctively analyse pairwise variation values for possible selection of multiple reference genes. In the present study, a combination of two, three, four, five or six genes did not affect target gene normalisation in the three tissue types and integrated sample based on V_n_/V_n+1_ < 0.15, which was usually used as a cutoff value in geNorm pairwise variation analysis in previous studies^21,22,25^. However, across the seasons, V_2_/V_3_ values for spring, autumn and winter were < 0.15, whereas summer sample exhibited V_5_/V_6_ < 0.15, showing that combination of two reference genes is suggested for normalization of target gene expression in spring, autumn and winter, while five genes are needed in summer sample.

Regardless of the optimal number of reference genes suggested for accurate normalisation of target gene expression levels by geNorm pairwise analysis, V_2_/V_3_ is suggested as minimum number of reference genes; therefore, at least two genes are required as an internal control when all V_n_/V_n+1_ values are < 0.15^21,29^. However, in order to reduce the financial and technical burden in experiments, the selection of one reference gene might be suitable if target gene expression levels obtained with a single reference are not significantly different from those obtained with multiple reference genes^10,12^. In the present study, across seasons and tissue types, the expression levels of *CAT* with normalisation by either *EIF* or *RPN2* were found to be significantly higher than those normalised with *ACT*, *EF1*, *RPS5*, *RPS18* or *GAPDH.* In addition, the C_q_ values of *EIF* and *RPN2* were relatively higher than those of the other five genes. However, across conditions, expression levels of *CAT* with *ACT*, *EF1*, *RPS5*, *RPS18* or *GAPDH* normalisation were statistically similar to those with multiple reference gene combinations. This suggests that single reference gene selection of *ACT*, *EF1*, *RPS5*, *RPS18* or *GAPDH* could be a possible alternative to a combination of multiple reference genes, despite geNorm pairwise analysis suggesting that multiple genes should be used based on V_n_/V_n+1_ values. Therefore, when our analyses are taken together, each of *ACT*, *EF1*, *RPS5*, *RPS18* and *GAPDH* is suggested to be suitable as reference gene for qRT-PCR analysis. Among these five genes, although other analysis revealed that expression stability values of all five genes were below the criteria, SD values of *ACT* and *EF1* were over 1.0, the cutoff line in BestKeeper. Therefore, in conclusion, all the stability values of *RPS5*, *RPS18* and *GAPDH* were below the cutoff values in each of the analysis methods used. Thus, we suggest that *RPS5*, *RPS18* and *GAPDH* are the most appropriate reference gene for accurate normalisation of target gene expression in honey bee samples prepared from various tissues and seasons.

## Methods

### Sample preparation and total RNA extraction

The honey bee colonies used in this study (an Italian hybrid) were maintained in the experimental apiary of College of Ecology and Environmental Science, Kyunpook National University, Sangju, Gyeongsangbuk-do, Rep. of Korea (36° 22′ 24. 41″ N, 128° 08′ 24.24″ E). Nurse bees were collected from three different colonies in spring (March 28, 2018), summer (June 27, 2017) and autumn (September 28, 2017) based on the ages and behaviours of bees; however, nurse bees were obtained from the central region of the colony in winter (December 27, 2017), following the previous study^5^. The collected nurses were immediately frozen with liquid nitrogen and stored at − 70 °C until RNA extraction.

For tissue analysis, the head, thorax and abdomen were separated from five bees and pooled as a single replication in a tube containing TRI reagent. RNA samples were prepared from three biological replicates. Each tissue sample was completely homogenised with a bullet blender and total RNA was extracted using the Direct-zol RNA Miniprep Plus kit (ZYMO RESEARCH, Irvine, CA, USA). Samples were treated with DNase I during the RNA extraction procedure to eliminate genomic DNA contamination following the manufacturer’s protocol^10^. The purity and quantity of the extracted RNAs were measured in triplicate using a SpectraMax QuickDrop spectrophotometer. The prepared RNA was then stored at − 70 °C until further use.

### Primer design and cloning

The sequence information of seven genes was obtained from the NCBI database (https://www.ncbi.nlm.nih.gov) and primers for the seven reference genes and the target gene were designed, following the previous study^7^. The primer sets for *EF1*, *RPS18* and *CAT* were designed based on two different exons to amplify genomic DNA containing introns if samples were contaminated by genomic DNA; therefore, they produced larger PCR products (Table 1). Amplification specificity and the lack of genomic DNA contamination were confirmed with gel electrophoresis.

For subcloning, total RNA was used as a template for the reverse transcription PCR reaction with a DiaStar OneStep RT-PCR kit (SOLGENT, Daejeon, Korea) using the following protocol: 50 °C for 30 min; 95 °C for 15 min; 35 cycles of 95 °C for 20 s, 58 °C for 40 s and 72 °C for 30 s; and 5 min at 72 °C. The gene-specific primers were used for each gene amplification (Table 1). They were then subcloned into the pGEM-T easy vector (PROMEGA, Madison, MU, USA). The plasmid-containing positive inserts were submitted for sequencing reactions using the M13 universal primers with an ABI PRISM 3730XL analyser.

### Quantitative real-time PCR

For cDNA synthesis, the amount of total RNA was standardised to 1 μg. The first strand of cDNA was synthesised with total RNA, extracted from different tissues over the four seasons using ReverTra Ace reverse transcriptase (TOYOBO, Osaka, Japan), by priming with oligo (dT) following the manufacturer’s protocol.

For the qRT-PCR assay, we used the CFX Connect Real-Time PCR detection system (BIO-RAD, Hercules, CA, USA) with CYBR GREEN methodology. The PCR efficiency of each gene was calculated from the given slope after running standard curves generated with four points of twofold serial dilutions of cDNA using the following formula: E = 2^−1/slope^. qRT-PCR reactions were performed in duplicate (technical replicates) using the following protocol: 95 °C for 1 min; and then 40 cycles of 95 °C for 15 s, 58 °C for 15 s and 72 °C for 30 s. Quantification cycle (C_q_) values of the seven candidate reference genes and the target gene (*CAT*) were obtained at the same fluorescence threshold (0.1).

To validate the selected reference genes after determination of their gene stabilities, the expression level of the target gene (*CAT*) was analysed. The C_q_ values for reference genes and *CAT* were obtained for each sample and then normalised by a relative quantification method adapted from the original concept of 2^−ΔΔCq^^30^. Reference genes were selected based on the stability rank of genes analysed by geNorm (Fig. 2) when multiple references were used for normalisation and the mean C_q_ value was used for analysis.

### Data analysis

The C_q_ distribution of genes across various seasons and tissues was analysed with Origin Pro 9.0 and the AM, SD and CV values were obtained (CV = SD/AM).

To evaluate the expression stability of the seven candidate reference genes, three freely-available software programs, namely NormFinder (version 0.953)^20^, BestKeeper (version 1)^31^ and geNorm (version 3.1)^25^, were used in this study. NormFinder calculates the stability values of each candidate gene based on the overall variation to evaluate the systematic error introduced for gene normalisation, wherein lower stability values indicate more stable genes^20^. BestKeeper determines the suitable reference genes by calculating the geometric mean of the genes’ C_q_ values and then the SD: lower SD values signify more stable genes. BestKeeper also calculates the correlation (R^2^) of each candidate gene with other genes. Thereafter, highly correlated candidate genes are combined to evaluate P values. Based on the results of BestKeeper, the candidate genes with relatively high R^2^ values but low SDs, CVs and P values are considered to be more stable genes. The geNorm automatically calculates an M value for each putative reference gene based on the geometric mean of all studied genes: more stable genes are indicated by lower M values. In addition, geNorm compares the pairwise variation (V) of a gene with the other genes; pairwise variation (V_n_/V_n+1_) is calculated to estimate the optimal number of reference genes required for accurate normalisation. A pairwise variation value below 0.15 indicates that an additional reference gene does not improve normalisation of target gene expression levels^25^.

SPSS for Windows (version 23.0) was used for statistical analysis of *CAT* expression (Fig. 5). The expression patterns of *CAT* among the four seasons and three tissue types, normalised with a single gene or with multiple reference genes, were statistically analysed using a one-way ANOVA followed by Tukey’s multiple comparison post-hoc test (Fig. 5A–H).

## Supplementary information

Supplementary Information.
