# Supplementary material for: Reference gene selection for qRT-PCR analysis of season- and tissue-specific gene expression profiles in the honey bee Apis mellifera
Source: Sci Rep. 2020 Aug 18;10:13935. doi: 10.1038/s41598-020-70965-4 (PMC7435199; doi:10.1038/s41598-020-70965-4)
Supplement: Supplementary file 1 — Supplementary Information. [file 41598_2020_70965_MOESM1_ESM.pdf]

Supplementary Information

Reference gene selection for qRT-PCR analysis of season- and tissue-specific gene expression profiles in the honey bee *Apis mellifera*

Ji Hyang Jeon[1], KyungHwan Moon[2], YeongHo Kim[2], and Young Ho Kim[1,2,*]

[1]Department of Applied Biology, Kyungpook National University, Sangju, Gyeongbuk, Republic of Korea

[2]Department of Ecological Science, Kyungpook National University, Sangju, Gyeongbuk, Republic of Korea

*****Corresponding author, yhkim05@knu.ac.kr (YHK)

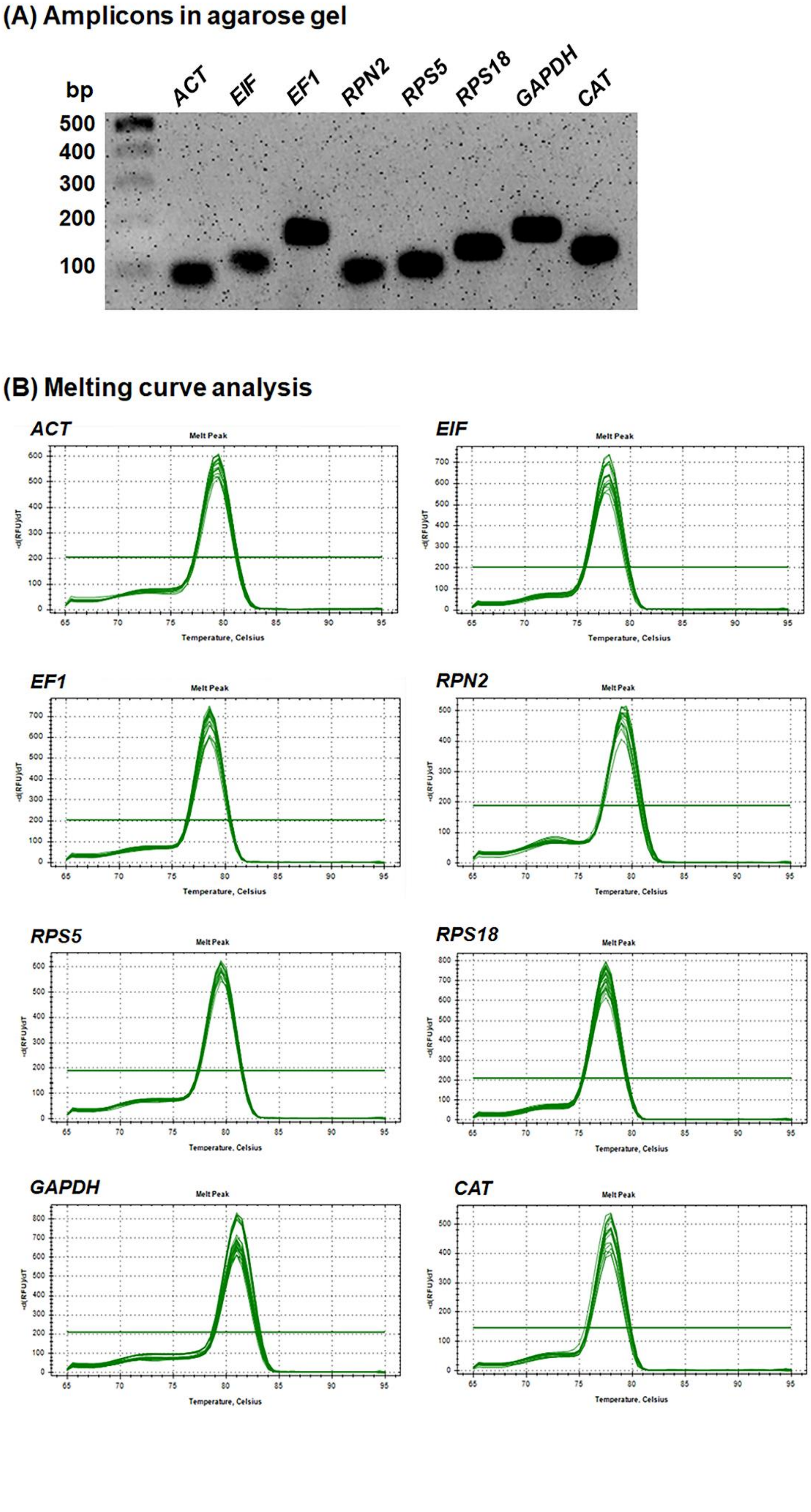
(A) Amplicons in agarose gel
bp
500
400
300
200
100
ACT
EIF
EF1
RPN2
RPS5
RPS18
GAPDH
CAT
(B) Melting curve analysis
ACT
EIF
EF1
RPN2
RPS5
RPS18
GAPDH
CAT
Melt Peak
-d(RFU)/dT
Temperature, Celsius

Supplementary figure 1. Specificity of qRT-PCR amplification. PCR products amplified with primers of seven candidate reference genes and a target gene (*CAT*) were visualized in 1% agarose gel (A). Melting curves for reference genes and *CAT* were analyzed by real-time PCR detection system (B).
